# A System of Medicine

**Published:** 1909-03

**Authors:** 


### Reviews of Books.

**A System of Medicine.** By Many Writers. Edited by Sir CLIFFORD ALLBUTT, K.C.B., M.A., M.D., and HUMPHRY DAVY ROLLESTON, M.A., M.D. Vol. IV, Part I. Pp. xiv, 764. London: Macmillan & Co. Limited. 1908.

Volume IV of the original *System* has now been divided into two. Twenty authors contribute some forty chapters on diseases of the Liver, Pancreas, Ductless Glands and Kidney.

Under the heading "Diseases of the Liver" there appears an entirely new article by Dr. Arthur Keith on "Hepatoptosis." Glénard, in 1872, found this condition in 20 per cent. of some 3,500 cases presenting themselves for treatment at Vichy. Many of these are incorrectly so classified, and are called partial only, whilst the true hepatoptosis is never an isolated condition, but always is part of a general sinking of the viscera, the condition now well known as visceroptosis (Vol. III, p. 860).

Another new article, that on "Biliary Cirrhosis," has been contributed by Dr. Morley Fletcher, and in place of the previous article by Dr. Fitz, of Boston, U.S.A., new articles on "Diseases of the Pancreas" have been written by Dr. Bosanquet and Dr. Newton Pitt. The authors do not write with any enthusiasm on the results of Cammidge's test, which they say has not given good results excepting in the hands of its inventor.

The article dealing with the "Thyroid Gland" is enriched by the addition of an entirely new account of myxœdema by Dr. George R. Murray, and the account of exophthalmic goitre by

Dr. Hector Mackenzie has been re-written. It takes a long time to establish any new treatment on a satisfactory basis ; accordingly treatment by means of the fresh milk of dethyroidised goats, or by the dried milk preparation, called Rodagen, are as yet only in the experimental stage, and hence there is still scope for the surgeon, although even in the hands of such a skilful surgeon as Kocher the operation is attended with considerable risk to life, and should not be lightly undertaken until every other means has failed to produce amelioration.

The volume concludes with over two hundred pages on "Diseases of the Kidney," a new article on "Nephritis" by Professor J. Rose Bradford taking the place of the previous one by Dr. Dickinson. It will be seen that this volume is to a great extent new, and not merely a reprint of the old edition. A very complete index by Dr. Jex Blake adds greatly to the utility of the work.

